# Modeling for extraction of oil from walnut and sesame using batch flow cold press oil extraction system

**DOI:** 10.1002/fsn3.2773

**Published:** 2022-02-22

**Authors:** Pedram Ghiasi, Omid Sohrabi, Edris Rahmati, Gholamhassan Najafi, Mazlan Mohamed, Azim Ghasemnezhad

**Affiliations:** ^1^ Department of Biosystems Engineering Tarbiat Modares University (TMU) Tehran Iran; ^2^ Department of Horticultural Science University of Guilan Guilan Iran; ^3^ Advanced Material Research Cluster Faculty of Bioengineering and Technology University of Malaysia Kelantan Jeli Kelantan Malaysia; ^4^ 123290 Department of Horticulture Faculty of Plant Production Gorgan University of Agricultural Sciences and Natural Resources Gorgan Iran

**Keywords:** cold press, oil extraction, oil pressure, optimization, regression model

## Abstract

In this study, a batch flow oil extraction system was used for extraction of oil from walnut (*Juglans regia* L.) and Sesamum (*Indicum sesame*). Sample mass (g), applied pressure (MPa), and processing temperature of oil (°C) were selected as independent variables and oil extraction mass percentage and oil analysis as dependent variables. Response surface methodology was employed for conducting statistical analysis, modeling, and data optimization. The results revealed that the highest percentage of oil extraction for walnut was obtained at a pressure of 10.5 MPa, a temperature of 31.5°C, and a sample weight of 8 g, with a value of 25.36%. Also, the highest percentage of oil extraction for Sesamum was obtained at the pressure of 13.88 MPa, the temperature of 31.5°C, and a sample value of 20 g with a value of 22.4%. Optimal level of independent variables for walnut and sesame were 8.03 g, 10.41 MPa, and 27.37°C; 20 g, 13.88 MPa, and 27°C, respectively. In this optimum condition, the amount of sesame and walnut peroxide was 10 ± 0.03 and 1.9 ± 0.07 (meq O2/kg), respectively. Likewise, the amount of acid for sesame and walnut was 1.53 ± 0.05 and 0.06 ± 0.02 g/%, separately. Linoleic acid (42.7–51.15), oleic acid (38.6–24.03), palmitic acid (10.87–8.21), and stearic acid (5.5–3.39) were the most common fatty acid components in sesame and walnuts, respectively.

## Graphical abstract

A new cold press oil extraction system was employed for extracting the oil from oilseeds (walnut and sesame) in the low volume. Sample mass (g), applied pressure (MPa), and processing temperature of oil (°C) were selected as independent variables. Response surface methodology was employed for statistical analysis and data optimization. Optimal level of independent variables for walnut and sesame were 8.03 g, 10.41 MPa, and 27.37°C; 20 g, 13.88 MPa, and 27°C, respectively.

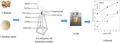

## 1 INTRODUCTION

Oil extraction from oilseeds is highly appreciated in various industries and therein, different methods have been employed depending on how the extracted oil may be used later on (Deli et al., 2011). Mechanical and chemical (using a solvent) extraction methods are commonly applied to extract oil from oilseeds (Mridula et al., 2020). The maximum efficiency of extraction using mechanical methods has been reported to be around 80%, but higher using solvent methods. However, chemical methods for extracting oil are often costly and there is a risk of ignition and explosion if the process comes in contact with fire. Also, the high temperature required for oil extraction has a negative effect on the biochemical profile of edible oils (Ajibola et al., 2002). Currently, mechanical procedures for oil extraction are gaining popularity because they are a simple and safe method with fewer steps, and the extracted oil can be processed quickly (Oyinlola et al., 2004; Pradhan et al., 2011). Mechanical methods fall into two groups of hot press and cold press. In hot‐press method, despite the higher percentage of oil extraction, the effect of temperature on the degradation of antioxidant components in the product is clearly visible (Ouchbani et al., 2021). Cold press is a method with the highest safety for extracting oil from oilseeds. It has been universally used in recent years (Azadmard‐Damirchi et al., 2010; Siger et al., 2008; Willems et al., 2008). According to this method, the extraction percentage is a function of various factors, such as extraction temperature, product moisture, pressure on the product surface, mass of sample, etc. Cold press has long been studied as rotary press, rolling press, and hydraulic press in various fields, but few studies have been dedicated to investigate important parameters affecting oil extraction efficiency (Aguilera & Stanley, 1999; Bako et al., 2020; Bamgboye & Adejumo, 2007; Chapuis et al., 2014). In order to maximize the oil extraction percentage and keep the minimum oil in the pulp, it is of remarkable importance to control the variables during the process, so that the uncontrolled variable will result in low efficiency and poor quality of the extracted oil (Bamgboye & Adejumo, 2011). Therefore, finding the relationship between independent variables of the extraction system and its effect on the percentage of oil extraction regarding different oilseeds can play an important role in designing and optimizing oil extraction systems. The oil extraction model, on the other hand, is regarded as one of the most important mechanical properties of an oilseed. To provide an appropriate regression model for oil extraction, various factors must always be considered to obtain a valid model with a proper generalization power.

Walnut with the scientific name (*Juglans regia* L) is one of the main oilseeds that is used all over the world due to its nutritional and medicinal properties. Depending on the cultivar, the percentage of moisture, and other environmental factors, the amount of walnut oil has been reported from 40% to 70% (Labuckas et al., 2014; Lachman et al., 2010; Martínez et al., 2008). Sesame (*Sesamum indicum* L.) is an annual, self‐pollinating plant belonging to the Pedaliaceae family that is cultivated in tropical and subtropical regions. Sesame seeds are of high nutritional and economic properties due to the high oil content (28%–59%) (Martínez et al., 2017); (Khazaei & Mohammadi, 2009; Rajeswari et al., 2010; Shenoy & Kalagudi, 2005; Shyu & Hwang, 2002; Willems et al., 2008). Numerous studies have been conducted with respect to the process of oil extraction from oilseeds using cold‐press method (Bako et al., 2020; Bogaert et al., 2018; Chouaibi et al., 2019; Huang et al., 2019). A study investigated the effect of screw rotation speed (40 and 70 rpm), temperature (90 and 120°C), and screw diameter (10 and 15 mm) on the yield of grape seed oil. The results showed that the highest percentage of oil (73%) was obtained at 40 rpm screw rotation speed, 15 mm screw diameter, and 90 ° temperature pretreatment. Also, based on the proposed regression model, they reported that the higher the seed moisture, the lower the oil extraction (Rombaut et al., 2015).

Bogaert et al. (2018) investigated the effect of screw rotation speed (0 to 18.2 rpm) on Canola and flax oil yield. The results revealed that increasing the screw rotation speed reduces the oil extraction performance. For this purpose, with increasing the screw rotation speed from 1.2 to 18.2, the oil yield decreased by 5% (Bogaert et al., 2018). In another study, in order to determine a regression model for oil extraction, the effect of screw rotation speed (15, 30, 45, 60, and 75 rpm), the amount of pressure applied (5, 10, 15, 20, and 25 MPa), and the feed speed 100, 200, 300, 400, and 500 g/min on oil extraction of African oil bean (*Pentaclethra macrophylla* Benth) were studied subsequently. The result of the regression polynomial equation was closely correlated with the experimental results (*R* ^2^ = 0.87). Based on the polynomial model presented in this experiment, optimal extraction was achieved at 45 rpm screw rotation speed, 20 MPa pressure, and feed rate of 300 g/min. Oil extraction increased with increasing pressure applied at a certain speed, but oil extraction decreased with high rotation speed. Also, the percentage of oil extraction at constant pressure decreased with increasing the feed rate (Ogunlade & Aremu, 2020). Martínez et al. (2008) utilized a screw press to extract walnut oil, followed by the supercritical fluid carbon dioxide. The highest oil extraction efficiency was obtained in moisture content of 7.5% and temperature of 50°C with 89.3%. In the moisture content of 7.5%, increasing the temperature from 50 to 70°C has led to a decrease in extraction efficiency. Increasing the temperature induces higher frictional resistance and consequently overheating of the material, so the pressure applied in these conditions would not be suitable for extraction (Martinez et al., 2008).

However, little research has been done with regard to the extraction of walnut and sesame oil using cold press mechanical system. Furthermore, no data on the effect of various process parameters on extraction efficiency, oil profile analysis, modeling, and process optimization have been reported. Therefore, in the present study, a batch flow cold press oil extraction system was developed. This system's key features include portability, low extraction time, and suitability for low‐volume samples. The effect of pressure applied, processing temperature of oil (microwave pretreatment), and mass of input material on the physicochemical properties of walnut seeds (*Juglans regia* L.) and sesame (*Sesamum Indicum*) was investigated to evaluate the designed system. Finally, the optimal level of product temperature, pressure applied, and mass of input materials for the optimal amount of oil extraction percentage were introduced, along with oil analysis.

## 2 MATERIALS AND METHODS

### 2.1 Sample preparation

Walnut with local genotype and local sesame mass was purchased from Kermanshah‐Iran local market. The removal process of sample impurities was conducted, thereafter, the samples were poured into polyethylene bags and kept at 23 ± 2℃. The initial moisture content of walnuts and sesame was 15% and 20%, respectively. According to Soxhlet extraction method, the amount of oil in walnut and sesame seeds was measured and then after reported 48 and 42%, respectively (Ghasemnezhad & Honermeier, 2007).

### 2.2 Mechanical cold press system

**FIGURE 1:**
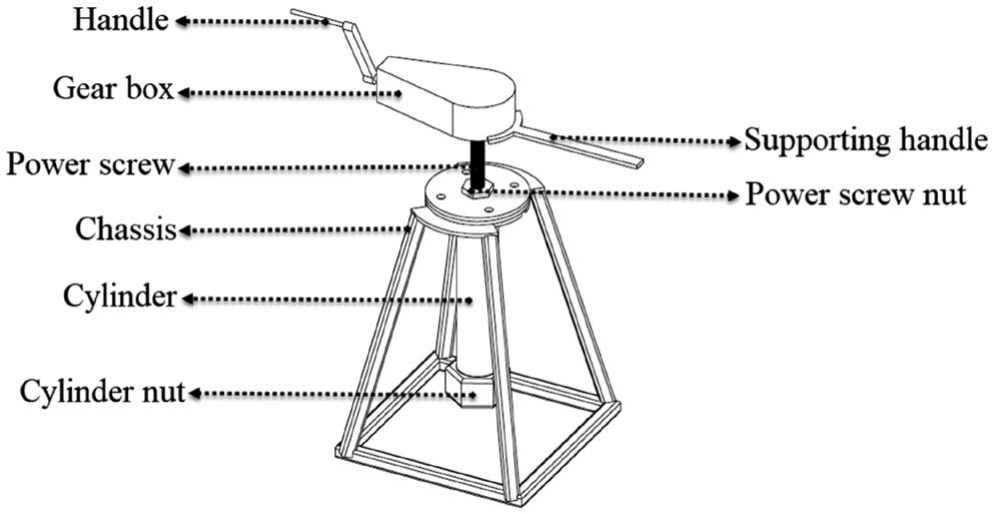
Schematic view of a fabricated mechanical press system

A mechanical cold press system was designed and then fabricated for oil extraction operations. The system was made up of the cylinder, piston, guide ring, power screw, gearbox, chassis, and screw rotation handle. The schematic illustration of the system is shown in Figure 1. The total stroke of the cylinder and piston and its diameter are 250 and 40 mm, respectively, which can compress a maximum of 314 mm^3^ of the sample. In order to prevent piston wear with the cylinder body, two guide rings were placed around the piston to seal on the piston and cylinder. A power screw with the pitch of 2 mm, length of 350 mm, and outer diameter of 20 mm was used, which was finally attached to the piston by a round bottom bearing. The 1:4 tapered gearbox was mounted on top of the power screw in order to provide more torque for applying the required pressure to the product. In order to extract the oil, a multi‐hole nut was designed and then utilized at the end of the cylinder. The 304‐grade stainless steel was selected for the material of the cylinder, piston and cylinder nut, and ASTM A36 steel for fabricating other parts of the system. This system's unique features included: portability, suitability for low‐volume samples, and a short extraction time.

### 2.3 Pressure and temperature measurement

**FIGURE 2:**
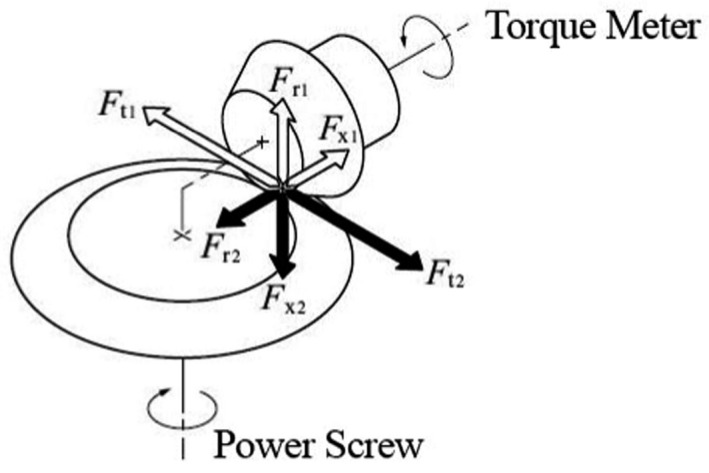
Schematic view of analog torque meter used in this study

After placing the material in the cylinder chamber, the cylinder screw was closed, and the piston handle was turned close to the material to press the material input. The rotation of the supporting handle continues until the oil will come out of the holes of the cylinder nut. The maximum torque applied was taken into account to calculate the amount of pressure. An analog torque meter (hazet 6000‐1 ct torque) with the capacity to measure 1 to 40 Nm was used to calculate the amount of torque applied. After measuring the torque applied to the handle, having the diameter of the gears in the gearbox, the amount of torque applied to the power screw was calculated. Figure 2 shows the gearbox force distribution.

Equations 1 and 2 were used to calculate the amount of force applied to the piston.

(1) $$T=\frac{F\;d_{m}}{2}\left(\frac{\pi \mu d_{m}‐1}{\pi d_{m}+\mu l}\right)$$

(2) l=nP

After calculating the force applied by the power screw to the piston, the amount of force applied by the piston to the material inside the cylinder was calculated via Equation 3.

(3) $$P=\frac{F}{A}$$

To increase the temperature of the samples (microwave pretreatment), a certain total of the sample was poured into a Pyrex Petridis and placed in the microwave (Model: Samsung M945 Microwave, 1000W). The samples were treated at 1000 watts for three times (60, 90, and 120 s). An optical thermometer (Model: RAYST6LXG) was used to calculate the surface temperature of sesame and walnut seeds.

### 2.4 Oil extraction efficiency and percentage analysis

Before each test, the amount of material entering the system was measured by a scale with an accuracy of 0.001 g (PS 1000.R1) and placed in the extractor cylinder chamber. After applying the desired torque, the sample was held for 60 s to completely remove the extracted oil and reach a steady state. After oil extraction process, the sum of pulp in the chamber of the machine was weighed again and the percentage of oil extracted according to Equation 4 was reported. It should be noted that all the extracted oil did not leave the chamber because part of the oil stuck to the cylinder wall, or remained in the space between the nut and the cylinder as the loss of the system.

(4) $$E=(1‐\frac{w_{t}‐w_{m}}{w_{t}})\times 100$$

Oil extraction efficiency is proportional to the percentage of total oil of seeds and the method recruited for oil extraction, which can be calculated based on Equation 5.

(5) $$\eta =\frac{E}{E_{t}}\times 100$$

### 2.5 Analysis of oil extract

#### 2.5.1 Peroxide value

To determine the peroxide values, 5 g of oil was mixed with 30 ml of acetic acid chloroform solution and homogenized with a stirrer. It was then treated with 0.5 ml of potassium iodide solution. Thirty milliliters of distilled water was added after 1 min and titrated with 0.01 N sodium thiosulfate. During the titration, the solution was shaken to release iodine from the chromium layer (Horwitz, 2002).

#### 2.5.2 Acidic value

To calculate the acid value, 3 g of oil was dissolved in 20 ml of ethanol/ether solution (1:1). Following that, a few drops of phenolphthalein were added to the solution, which was then titrated with 0.1 N KOH. Titration through 0.2 N oxalic acid solution determined the normality of KOH solution (Abduh et al., 2013).

#### 2.5.3 Oil analysis

The fatty acids were prepared using a method developed by Savage et al. (1999). One microliter of oil prepared in gas chromatography (made in Iran, model CP‐Sil 88) was injected for this purpose as follows. A FID detector with a column length of 30 meters, a film thickness of 0.25 meters, and a hydrogen carrier gas were used in the device. Following injection of the sample into gas chromatography, a curve was drawn and the inhibition time of each fatty acid was compared to the standard fatty acid curve. The temperatures of the injector and detector were 220 and 250°C, respectively. The inhibition value for fatty acids was calculated using a standard diagram made from fatty acids.

### 2.6 Statistical analysis

In the current study, the response surface methodology and the Box–Behnken design were recruited to perform the design of the experiment. Response surface is a combination of mathematical and statistical method for constructing regression polynomial equation that simultaneously evaluates the effect of several independent variables, with the aim of providing optimal surface in order to achieve the desired value of the dependent variable (Breig & Luti, 2021). With a quadratic polynomial equation, the mathematical relationship between independent and dependent variables is expressed as follows:

(6) $$\begin{array}{cc} Y_{i}= & a_{0}+a_{1}X_{1}+a_{2}X_{2}+a_{3}X_{3}+a_{11}X_{1}^{2}+a_{22}X_{2}^{2}+a_{33}X_{3}^{2}\\ & +a_{12}X_{12}+a_{13}X_{13}+a_{23}X_{23} \end{array}$$

In this regard, *Y* is the dependent variable, *X*_1_, *X*_2_, and *X*_3_ are the independent variables, *a*_1_, *a*_2_, and *a*_3_ are the linear regression coefficients, *a*_11_, *a*_22_, and *a*_33_ are the quadratic regression coefficients, and *a*_12_, *a*_13_, and *a*_23_ are the regression coefficients with respect to the interaction effects.

In this study, the independent variables were sample temperature, pressure applied to the product surface, and the mass of input materials, while the dependent variables were the percentage of oil extraction and oil analysis.

In this regard, three levels considered including the product temperature, pressure applied, and the mass of input materials for walnuts (23, 31.5, and 40°C), (3.5, 7, and 10.5 MPa), and (8, 16, and 24 g); and for sesame (23, 31.5, and 40°C), (3.5, 8.7, and 13.9 MPa), and (20, 60, and 100 g), respectively. Statistical analysis of treatments was performed using Design Expert software (Design‐Expert^®^ Software Version 10). Modeling and process optimization were carried out following the analysis of variance and the significance of the regression model. Eventually, the proposed optimal conditions were experimentally validated via the response surface method.

## 3 RESULTS AND DISCUSSION

### 3.1 Experimental results for walnut

The percentage of oil extraction ranged from 13% to 25.36% and the oil extraction efficiency ranged from 30.9% to 60.38%. Under certain conditions, the highest percentage of oil extraction was obtained at the pressure of 10.5 MPa, the temperature of 31.5°C, and a sample value of 8 g with a value of 25.36%. The results of ANOVA along the regression coefficients for the percentage of walnut oil extraction are shown in Table 1. The Box–Behnken design proposes a quadratic regression model for the experimental data. According to this model, the input mass and applied pressure on materials have a significant effect on extraction percentage (*p* < .01). Furthermore, the simple and second‐order effects of oil processing temperature on oil extraction percentage were significant at the 5% level.

**TABLE 1:** ANOVA for variables studied in cold mechanical press system for walnut

Source	Sum of squares	*df*	Means of square	*F*‐value
Model	177.92	9	19.77	37.39**
*A*‐weight (g)	49.89	1	49.89	94.37**
*B*‐pressure (MPa)	69.19	1	69.19	130.87**
*C*‐temperature (°C)	2.64	1	2.64	4.99*
AB	0.25	1	0.25	0.47ns
AC	1.77	1	1.77	3.35ns
BC	1.18	1	1.18	2.22ns
*A* ^2^	45.10	1	45.10	85.31**
*B* ^2^	0.13	1	0.13	0.26ns
*C* ^2^	10.88	1	10.88	20.58*
Residual				
Lack of fit	2.35	3	0.78	0.16ns
Pure error	0.29	2	0.15	
*SD*	0.73			
C.V. %	3.88			

*significant value (*p* < .05) and **significant value (*p* < .01)

**FIGURE 3:**
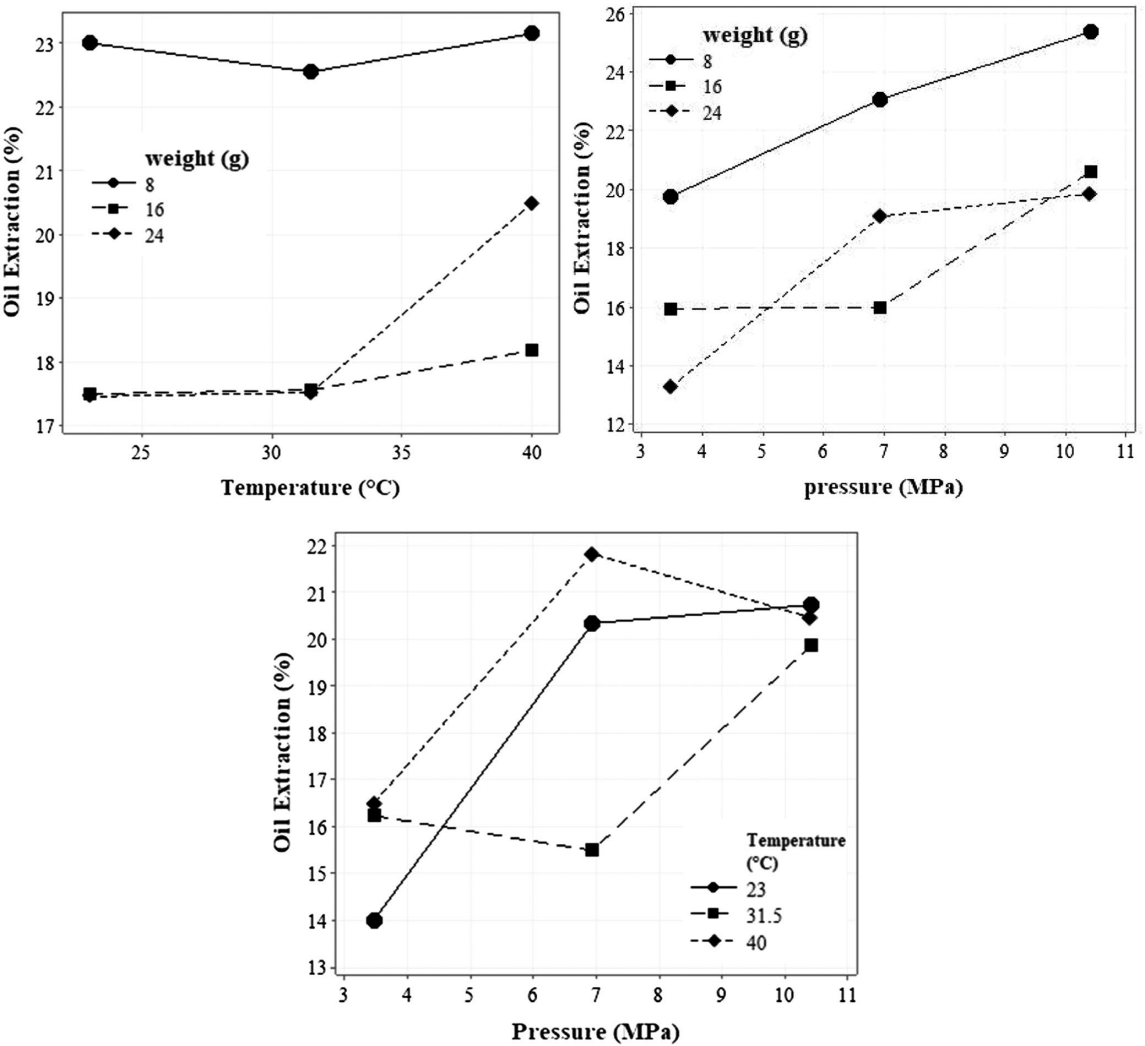
The effect of applied pressure, processing temperature of oil, and sample mass on the extraction efficiency of walnut oil

Figure 3 shows the effect of pressure applied to the product on the extraction percentage for different mass values. As can be seen, it rises as the pressure on the material increases and the amount of oil sample extracted decreases. It may be concluded that at a constant pressure, increasing the mass of the material causes less pressure on each element of the material and the amount of oil extracted also decreases with the decline of pressure applied to the element. Analogous results have been reported by roselle and moringa (Bamgboye & Adejumo, 2011; Fakayode & Ajav, 2016). In these studies, increasing the pressure from 5 to 20 MPa has led to an increase in extraction efficiency. The application of high pressure (the pressure greater than 20 MPa) reduced the extraction efficiency. This is due to the closure of the internal cavities of the seeds at high pressure, so the optimal pressure must be reported for each product.

The effect of temperature on the percentage of oil extraction for different masses is shown in Figure 3. The general trend of changes in the percentage of oil extraction is increasing with the temperature increment, but considering the increase of temperature from 23 to 31.5°C and the mass of 8 g, the percentage of oil extraction has decreased from 23% to 22.55%. Generally, increasing the temperature leads to the increment of extraction efficiency; actually, because temperature gives rise to oil cell structure loss, increased oil fluidity, and protein coagulation (Fakayode & Ajav, 2016). These findings are visibly in line with other studies (Taghvaei et al., 2014, 2015; Uquiche et al., 2008).

### 3.2 Sesame seed

**FIGURE 4:**
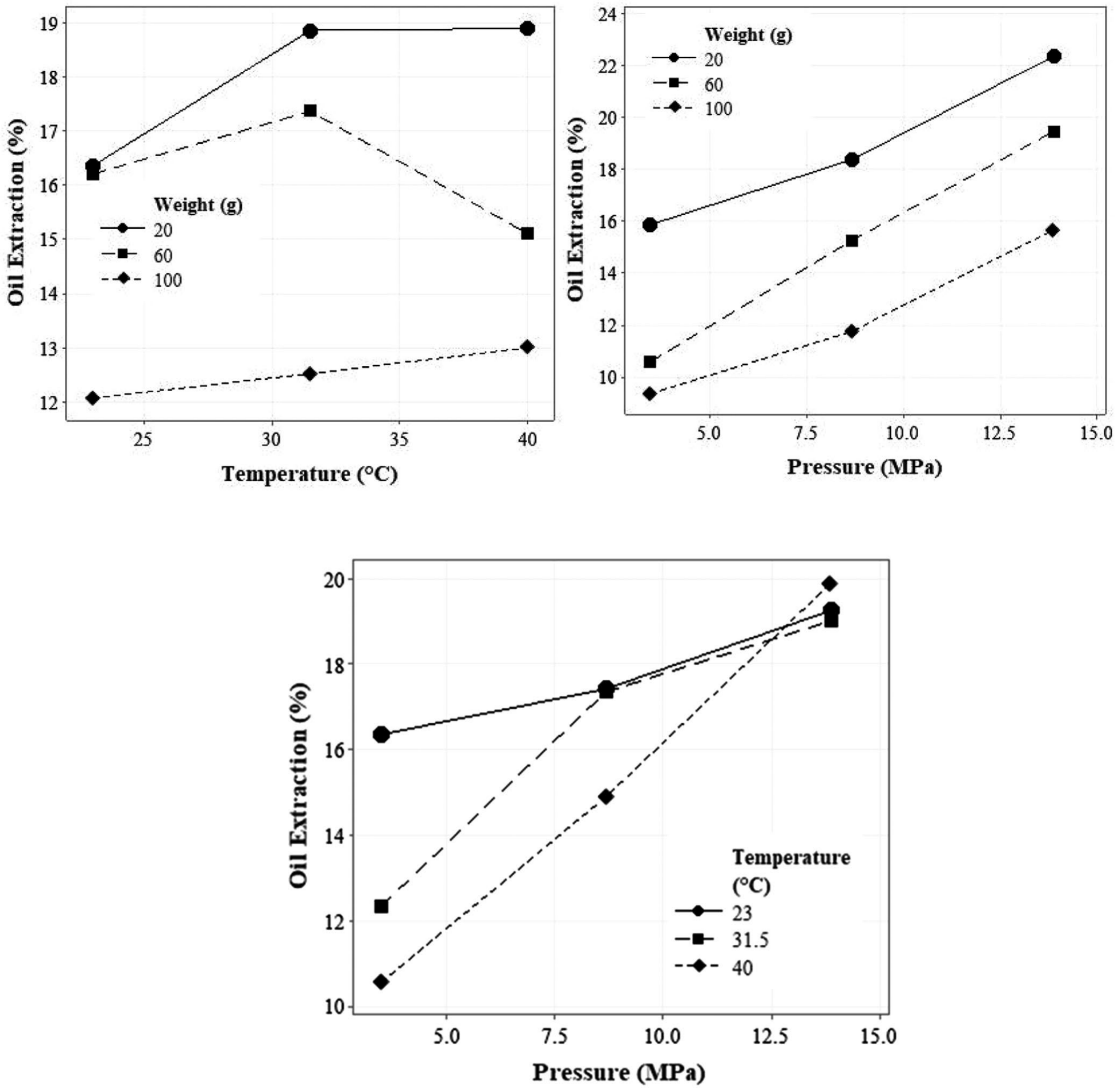
The effect of applied pressure, processing temperature of oil, and sample mass on the extraction efficiency of sesame oil

Table 2 shows the statistical analysis results for the variables tested in the cold mechanical press system for the percentage of sesame oil extraction. As a result, the simple effects of input mass and material pressure on oil extraction percentage are significant at the 1% level. Figure 4 illustrates the effect of pressure applied to the product on sesame extraction efficiency given different masses. With increasing the pressure on the materials and decreasing the mass, the percentage of oil extraction enlarged, since increasing the pressure causes damage to the sesame seed tissue, and the percentage of oil extraction increases during this process.

**TABLE 2:** ANOVA for variables studied in mechanical cold press system for sesame

Source	Sum of squares	*df*	Means of square	*F*‐value
Model	185.26	9	20.58	35.01**
*A*‐weight (g)	71.44	1	71.44	121.52**
*B*‐pressure (MPa)	101.75	1	101.75	173.06**
*C*‐temperature (°C)	0.078	1	0.078	0.13ns
AB	0.12	1	0.12	0.21ns
AC	1.72	1	1.72	2.93ns
BC	2.21	1	2.21	3.76ns
*A* ^2^	3.97	1	3.97	6.76ns
*B* ^2^	0.0091	1	0.0091	0.016ns
*C* ^2^	4.41	1	4.41	7.5ns
Residual				
Lack of fit	2.27	3	0.76	2.24ns
Pure error	0.67	2	0.34	
*SD*	1.12			
C.V. %	7.17			

**significant value (*p* < .01)

Martinez et al. investigated the effect of moisture content, pressing speed, and die restriction for optimal extraction of sesame oil with screw‐pressing. The maximum extraction efficiency was 12.3% for moisture content, with pressing speed of 20 rpm and restriction die of 4 mm with the value of 71.1% (Martinez et al., 2017). Willems et al. (2008) used a hydraulic press to investigate the effect of temperature, moisture content, seed content, and pressure on the yield of oilseeds (sesame, linseed, rapeseed, jatropha, and palm kernel). The oil content of linseed, rapeseed, and jatropha seeds were between 45% and 55%, and the oil content of sesame, cocoa, and dehulled jatropha seeds were from 70% to 75%. The results revealed that increasing the temperature and pressure had resulted in extraction efficiency while the sample size had no significant effect on the extraction efficiency.

Also, increasing the amount of moisture at a constant pressure and temperature led to a decrease in the compaction rate and density of the samples. Finally, it was reported that at 100°C and with an optimum humidity, climbing the pressure leads to increasing the extraction efficiency of all seeds (Willems et al., 2008). With increasing the inlet temperature, no significant effect was observed with respect to the extraction efficiency of sesame oil (*p* > .05). This is due to the fact that the applied temperature does not have the ability to destroy the cellular structure of plant tissue (Taghvaei et al., 2014). The cell membrane of oilseeds acts as a barrier to oil extraction in the cold press process. This flaw can be fixed with a microwave. Microwave increases porosity and improves oil extraction (Uquiche et al., 2008).

### 3.3 Results of regression modeling and optimization of experimental variables

Two coded regression polynomial equations were obtained to predict oil extraction efficiency using Equation (6). These regression polynomial equations were attained after analysis of variance using the response surface methodology to predict the dependent variables. These regression polynomial equations show the effect of independent variables, that is, the product temperature, the pressure applied to the product surface, and the mass of inputs on oil extraction efficiency in Equation (7) and (8), respectively. In these equations, the value and sign of each coefficient of the independent variables indicate the importance of the variable and the trend of changes with the percentage of oil extraction, respectively.

**FIGURE 5:**
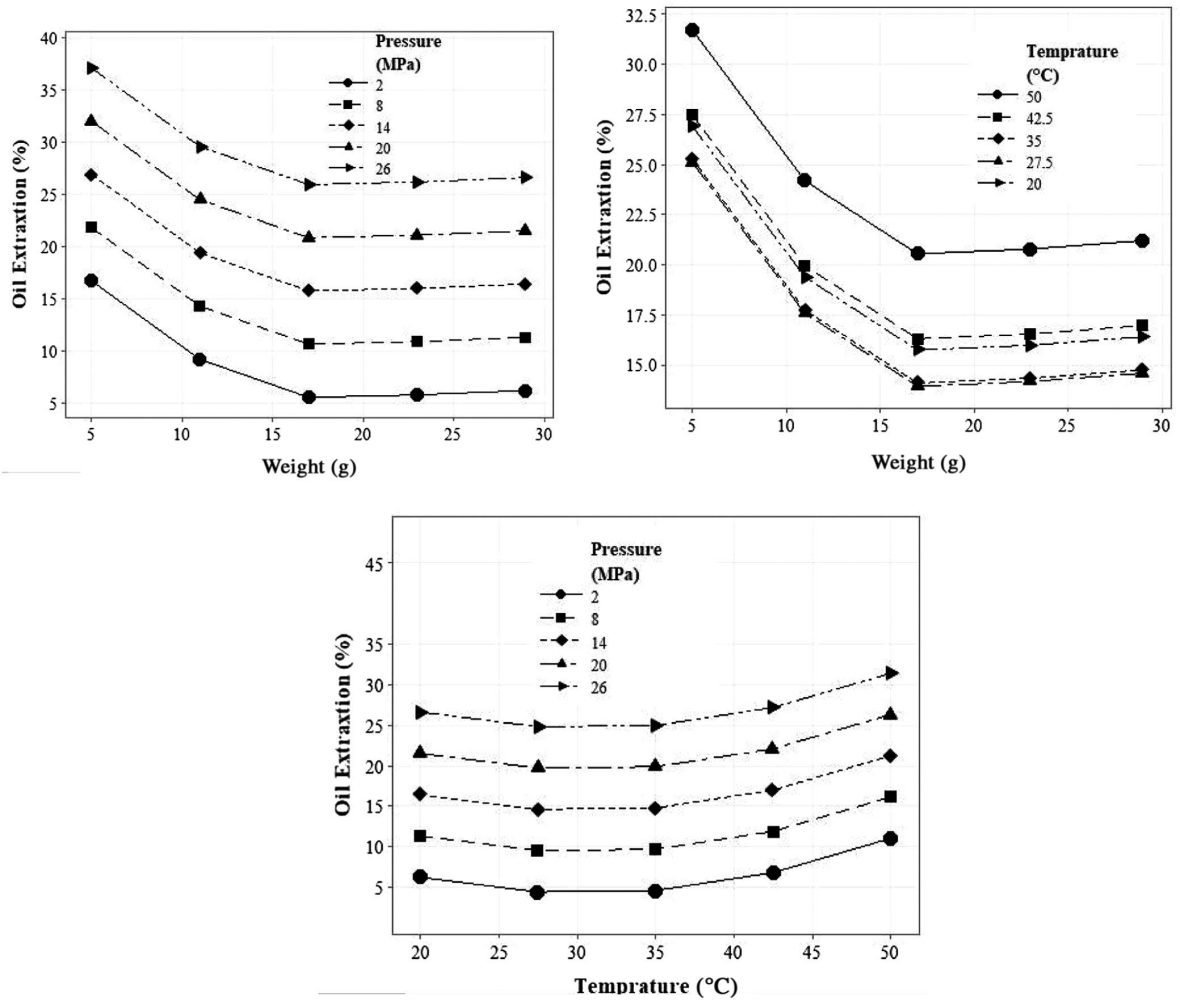
The effect of different parameters on the extraction efficiency of walnut oil

The coefficient of determination for walnut and sesame was 0.9669 and 0.9844, correspondingly, which implied the accuracy and predictability of the model. In addition, there is a high correlation between experimental and predicted data. Since only the simple effects of product temperature, pressure, and mass of input materials and second‐order effects of mass and processing temperature of oil on oil extraction percentage were significant, in the regression model these parameters were considered as effective variables. Considering the regression coefficients, the simple effects of weight and pressure had the most and least effect on the extraction efficiency of walnut oil, respectively. For A, the negative sign indicates the reverse trend of changes and the positive sign A^2^ designates that the trend is reversed for the mass of 19.63 g and a curved relationship was observed between the mass of the input material and the percentage of oil extraction. An increase in the percentage of oil extraction was observed with the mass of 29 g compared to the mass of 23 g in any given pressure. A positive coefficient of B indicates a direct relationship between the pressure applied to the material and the percentage of oil extraction. From coefficients C and C^2^, it can be seen that the relationship between material temperature and oil extraction percentage in the test interval could be plotted as a U‐shaped curve. The temperature at which the oil extraction percentage trend will be reversed is 39.44°C (Figure 5). As is in sight, the effect of temperature on the extraction efficiency is down to 39.44, but with the temperature passing through the point 39.44, the trend of oil extraction percentage straightens with temperature.

(7) $$E=50.26‐2.12A+0.85B+1.42C+0.054A^{2}+0.018C^{2}$$

*R* ^2^ =0.9669; $R_{\text{adjust}}^{2}$: 0.959.

Fakayode & Ajav, (2016) in a study on Moringa concluded that increasing the temperature from 50 to 80°C leads to increased extraction efficiency, but at high temperatures the yield decreases, as the high temperature gives rise to a decrease in moisture content and hardness. The resistance of the sample increases during the process of applying the pressure, which induces a decrease in the extraction efficiency of the sample (Fakayode & Ajav, 2016). In a study, Bakhshabadi et al. (2017) investigated the effect of microwave pretreatment (microwave power and processing time) for optimal extraction of cumin seed oil. The results showed that increasing the microwave power and processing time effectuates improved extraction efficiency. Microwaves cause plant cells to deform, disintegrate, and destroy membranes. Changing the structure of the product and thus destroying the membrane and cell wall of the product effectively impacts on the extraction efficiency. This is so, because in the initial state, the cellular structure of the plant is in the form of a band (Aguilera & Stanley, 1999; Bakhshabadi et al., 2017). However, Deli et al. (2011) studied the effect of different parameters on extraction efficiency (*Nigella sativa* L seeds). The findings exposed that increasing the temperature from 50 to 100°C reduces the extraction efficiency. This is due to the change in product moisture content and internal seed structure during the heating process (Ogunsina et al., 2008). Heat strengthens the bond between proteins and oils within the material's structure, preventing the oil from easily releasing from the seed (Deli et al., 2011). The model proposed in this study can predict the positive effect of temperature increase on oil extraction percentage up to 50°C, which is firmly in convergence with the results of the abovementioned studies. On the basis of the body of literature, increasing the temperature from 50°C requires experimental testing, so the oil extraction behavior at higher temperatures might be different.

**FIGURE 6:**
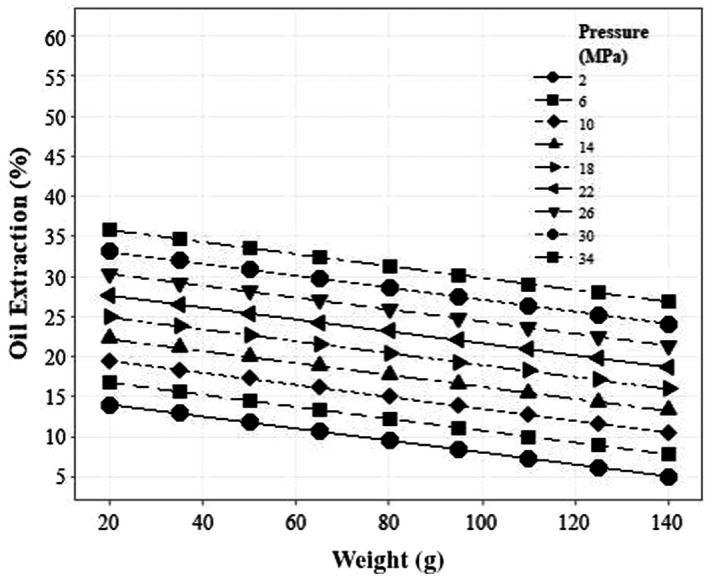
The effect of applied pressure and sample mass on the extraction efficiency of sesame oil

In the case of sesame, only the simple effects of input mass and the force applied to the product surface were significant regarding the percentage of oil extraction, so in the regression model, these parameters were considered effective variables. By comparing the regression coefficients, the simple effects of force and mass of the input materials had the most and the least effect on the sesame oil extraction efficiency, respectively. The trend of changes with respect to the force applied to the materials and mass of materials with oil extraction percentage is direct and inverse, respectively. Also, the trend of oil extraction percentage with two parameters of pressure to materials and material mass is linear. Figure 6 shows the linear trend of the applied pressure and the mass of the material. The model predicts that with the mass of 20 g and the pressure of 34 MPa, 35.75% of the total mass entering the chamber can be extracted with the efficiency of 89.88%.

(8) η=14.12‐0.0747A+0.68B

*R* ^2^ = 0.9844; $R_{\text{adjust}}^{2}$: 0.9067.

In order to achieve the optimal amount of oil extraction efficiency, the process was optimized based on experimental data using design expert software for both walnut and sesame products. Table 3 shows the optimal level of each independent variables and the predicted values for the percentage of the oil extraction. For walnuts, the sample mass of 8.03 g, the pressure of 10.41 MPa, and the processing temperature of 27.37°C led to the highest extraction efficiency with a desirability function of 1. Under these conditions, the highest extraction efficiency was 25.743%. In addition, for sesame, the sample mass of 20 g, the pressure of 13.88 MPa, and the processing temperature of 27°C led to the highest extraction efficiency with a desirability function of 0.98. Under these conditions, the highest extraction efficiency was 22.142%. The optimized sample mass, applied pressure, and processing temperature of oil were examined to evaluate the efficiency of regression models for forecasting and optimizing responses (Table 3). These results show validity of developed models for predicting and optimizing responses.

**TABLE 3:** Experimental and predicted values regarding the cold press system for walnut and sesame oil extraction

Responses	Experimental value	Predicted value	|(%) Relative error|
Oil extraction (%)‐Walnut	25.68	25.743	0.24
Oil extraction (%)‐Sesame	22.38	22.57	0.84

### 3.4 Physicochemical profiles of oil

#### 3.4.1 Peroxide value and acidic value

In the current study, the physicochemical profiles of two products at optimal points were investigated. The peroxide value indicates the degree of oxidation of unsaturated fatty acids and organoleptic properties of the oil. Acidity also specifies the amount of free fatty acids in the walnut oil. Sesame and walnut peroxide values in the optimal points were 10 ± 0.03 and 1.9 ± 0.07 (meq O2/kg), respectively. Also, the value of acid for sesame and walnut was 1.53 ± 0.05 and 0.06 ± 0.02 g/%), individually (Table 4). The results of physicochemical properties (peroxide and acidic values) of sesame and walnut oil were in line with previous research. However, various factors, including the oxidation temperature, the method used to extract the oil, and the type of fatty acids in the oil, determine the peroxide value (Chatrabnous et al., 2018; Mbaebie et al., 2010; Zanjani et al., 2020).

**TABLE 4:** Chemical parameters of sesame and walnut crude oil

Sample	Peroxide value (meq O2/kg)	Acidic number (g/%)
Sesame	10 ± 0.03	1.53 ± 0.05
Walnut	1.9 ± 0.07	0.06 ± 0.02

#### 3.4.2 Fatty acid composition

The composition of the fatty acid profile of sesame and walnut oil extracted using the system designed is shown in Table 5. The most common constituents of fatty acids in sesame and walnuts were linoleic acid (42.7–51.15), oleic acid (38.6–24.03), palmitic acid (10.87–8.21), and stearic acid (5.5–3.39), respectively. The percentage of sesame and walnut fatty acid compounds in this study was in alignment with results reported by other researchers (Elleuch et al., 2007; Ozcan, 2009). Fatty acid compounds are an important indicator of the nutritional value of oils extracted from oilseeds. Diversity in genetics, climate, oil processing methods, and harvesting conditions can all cause differences in the fatty acid composition of different sesame and walnut seed oils (Hassan, 2012).

**TABLE 5:** Fatty acid profile (%) of sesame and walnut crude oil

Fatty acid Composition	Sesame	Walnut
Palmitic acid (16:0)	10.87 ± 1.20	8.21 ± 1.60
Stearic acid (18:0)	5.5 ± 0.80	3.39 ± 0.89
Oleic acid (18:1)	38.6 ± 1.1	24.03 ± 0.83
Linoleic acid (18:2)	42.7 ± 1.4	51.15 ± 0.61

## 4 CONCLUSIONS

Because edible oils are widely used, their safety and shelf life are critical. As a result, new technologies must be developed in order to effectively extract oil while also minimizing oil quality losses. An oil extraction system based on the cold method for sesame and walnut oil extraction was developed in this study. The cold press system was used to successfully extract oil. The amount of extracted oil increased as the amount of applied pressure increased. Furthermore, the oil extraction efficiency increased through reducing the mass of the input materials at constant pressure. The highest oil extraction efficiency (22.4) was observed for sesame at the pressure of 13.88 MPa, the temperature of 31.5°C, and a sample value of 20 g and for walnut (25.36) at a pressure of 10.5 MPa, a temperature of 31.5°C, and a sample weight of 8 g. Peroxide and acidity of sesame and walnut were 10 ± 0.03, 1.9 ± 0.07 (meq O2/kg), 1.53 ± 0.05 and 0.06 ± 0.02 g/%, respectively. The most common fatty acid compounds in sesame and walnuts were linoleic acid (42.7–51.15), oleic acid (38.6–24.03), palmitic acid (10.87–8.21), and stearic acid (5.5–3.39), respectively. Based on the findings, the system developed in the current study could be used in the future to extract sesame and walnut oil without compromising quality parameters.

## CONFLICT OF INTEREST

The authors declare that they have no conflict of interest.

## ETHICAL APPROVAL

This study did not involve any human or animal testing.

## Data Availability

The data that support the findings of this study are available from the corresponding author upon reasonable request.
